# Decreased serum creatinine levels predict short survival in amyotrophic lateral sclerosis

**DOI:** 10.1002/acn3.51299

**Published:** 2021-01-15

**Authors:** Qi‐Fu Guo, Wei Hu, Liu‐Qing Xu, Hao Luo, Ning Wang, Qi‐Jie Zhang

**Affiliations:** ^1^ Department of Neurology Fujian Institute of Neurology The First Affiliated Hospital Fujian Medical University Fuzhou China; ^2^ Fujian Key Laboratory of Molecular Neurology Fujian Medical University Fuzhou China

## Abstract

**Objective:**

To explore the associations between serum creatinine and creatine kinase (CK) levels with survival in male and female ALS patients.

**Methods:**

A prospective cohort study was carried out including 346 ALS patients with repeated serum creatinine and CK measurements. Kaplan Meier analysis and multivariable Cox regression were used to perform survival analysis.

**Results:**

There were 218 male and 128 female patients, and the males had significantly higher baseline serum creatinine and CK levels than females. After multivariable Cox regression analysis, lower baseline serum creatinine levels were associated with a short survival in both male (≤61 *μ*mol/L, HR: 1.629; 95%CI: 1.168–2.273) and female ALS patients (≤52 *μ*mol/L, HR: 1.677; 95%CI: 1.042–2.699), whereas, the serum CK levels were not correlated with survival. Besides, creatinine levels were positively associated with ALSFRS‐R scores, and inversely with the decline rate of ALSFRS‐R per month. During follow‐up, serum creatinine levels tended to be decreased along with the disease progression, and the higher decline rate of creatinine per month (>1.5) showed significantly shorter survival, compared to the lower group (≤1.5) (30.0 months vs. 65.0 months, Chi square = 28.25, *P* < 0.0001).

**Interpretation:**

Serum creatinine could be a reliable and easily accessible prognostic chemical marker for ALS, and decreased baseline creatinine levels could predict a poor prognosis and a short survival in both male and female ALS patients.

## Introduction

Amyotrophic lateral sclerosis (ALS), also known as Lou Gehrig’s disease or motor neuron disease (MND), is a kind of adult‐onset and fatal neurodegenerative disorder featured by progressive loss of both upper and lower motor neurons.^1^ Clinically, it is characterized by male predominance, relentlessly progressive muscle weakness and atrophy, highly variable natural history, and a wide range of survival time after disease onset from 6 months to 50 years.^2^, ^3^ However, the underlying mechanisms of diverse disease progression rate have not been completely elucidated. Promisingly, several prognostic biomarker candidates have been received increasing attention recently, for instance, neurofilament heavy chain (NfH), neurofilament light chain (NfL), uric acid, creatine kinase (CK), creatinine, albumin, ferritin, and lipid metabolic profiles.^4^ Among these fluid‐based biochemical biomarkers, serum creatinine and CK, prominent metabolic products of muscle in human beings, could reflect the muscle atrophy and denervation in ALS, and advantageously, they are inexpensive and readily available in almost every neurological department.^5^, ^6^ The creatinine showed neurologically protective effects in ALS mice models, and significantly lower levels of serum creatinine were observed in ALS patients.^7^, ^8^, ^9^ However, the roles of serum creatinine and CK in the prediction of disease progression and survival were conflicting.^10^, ^11^, ^12^ Besides, the longitudinal alterations of serum creatinine and CK at the different stages of ALS were not clarified. In the present study, we described the serum creatinine and CK levels at baseline and different time points of follow‐up in a prospective cohort of 346 patients with ALS, and further explored the correlations between serum creatinine and CK with the survival.

## Methods

### Patients

All the ALS patients were enrolled serially from December 2014 to September 2019 in the Department of Neurology, First Affiliated Hospital of Fujian Medical University, Fuzhou, China. The diagnosis of ALS based on refers to the revised ALS Escorial criteria, and was confirmed by two professional neurologists.^13^ The ALS staging was assessed according to the King’s College staging system.^14^ The ALS patients additionally combined with renal disease were excluded from the analysis. Each enrolled patient was followed up every 3–6 months in person. For those patients at later stages, they were followed up by telephone. Besides, all the patients were followed up more than 12 months, and the last visit time was April 2020. This study was approved by the Ethics Board of First Affiliated Hospital of Fujian Medical University. Written informed consent was obtained from each participant.

### Clinical data collection

At the time of diagnosis (baseline), clinical profiles including demographic characteristics, age of onset, site of first symptom onset, body mass index (BMI), family history, medical history, neurophysiological tests and treatments were recorded. The time interval (month) between disease onset and an identified ALS diagnosis was defined as “diagnostic delay.” The level of motor function impairment was assessed by the revised Functional Rating Scale (ALSFRS‐R, range from 1 to 48 score). The disease progression rate was calculated by the linear change rate of ALSFRS‐R (ΔALSFRS‐R), which was calculated by the equation: ΔALSFRS‐R = (48 − ALSFRS‐R at baseline)/duration from symptom onset to baseline clinical assessment in months. “Use of riluzole” was defined as administration of riluzole for longer than 3 months, with a dose of 50 mg, twice per day. “Use of noninvasive positive pressure ventilation (NIPPV)” was defined as acceptance of NIPPV support for longer than 4 h per day. The combined primary outcome was death or tracheotomy. Peripheral blood sample was drawn after 8 h of overnight fasting, and the serum creatinine and CK levels were quantified in the Department of Laboratory, the First Affiliated Hospital of Fujian Medical University. For the patients whose serum creatinine or CK levels were measured in other medical centers with certified lab, we recorded the results that were measured within 3 months before the initial clinical assessment. The reported upper limit of normal serum creatinine ranged from 84 to 135 *μ*mol/L. The reported upper limit of normal serum CK ranged from 140 to 310 U/L.

### Statistical analysis

The statistical analysis was performed by the SPSS statistical software (version 17.0, Inc, IL, USA). For survival analysis, the variables were described as dichotomy, and the cut‐off values were identified using non‐commercial X‐tile software (version 3.6.1, Yale University, USA). Kaplan‐Meier curves for low or high creatinine/CK were drawn, and then tested their difference with a log‐rank test. Subsequently, the effect of low or high creatinine/CK on overall survival was assessed in Cox regression model (Backward Stepwise, Wald) and adjusted for the following covariates, including age of onset, site of onset, diagnostic delay, BMI, ALSFRS‐R score, progression rate (ΔALSFRS‐R), use of riluzole, use of NIPPV, and acceptance of PEG. Correlations were analyzed with the Spearman coefficient (*r_s_*). For CK, there were six missing data, and we replaced them with the median value (174.5 U/L) of the entire cohort. *P* value less than 0.05 was considered statistically significant.

## Results

### Clinical features of ALS patients

**Figure 1:**
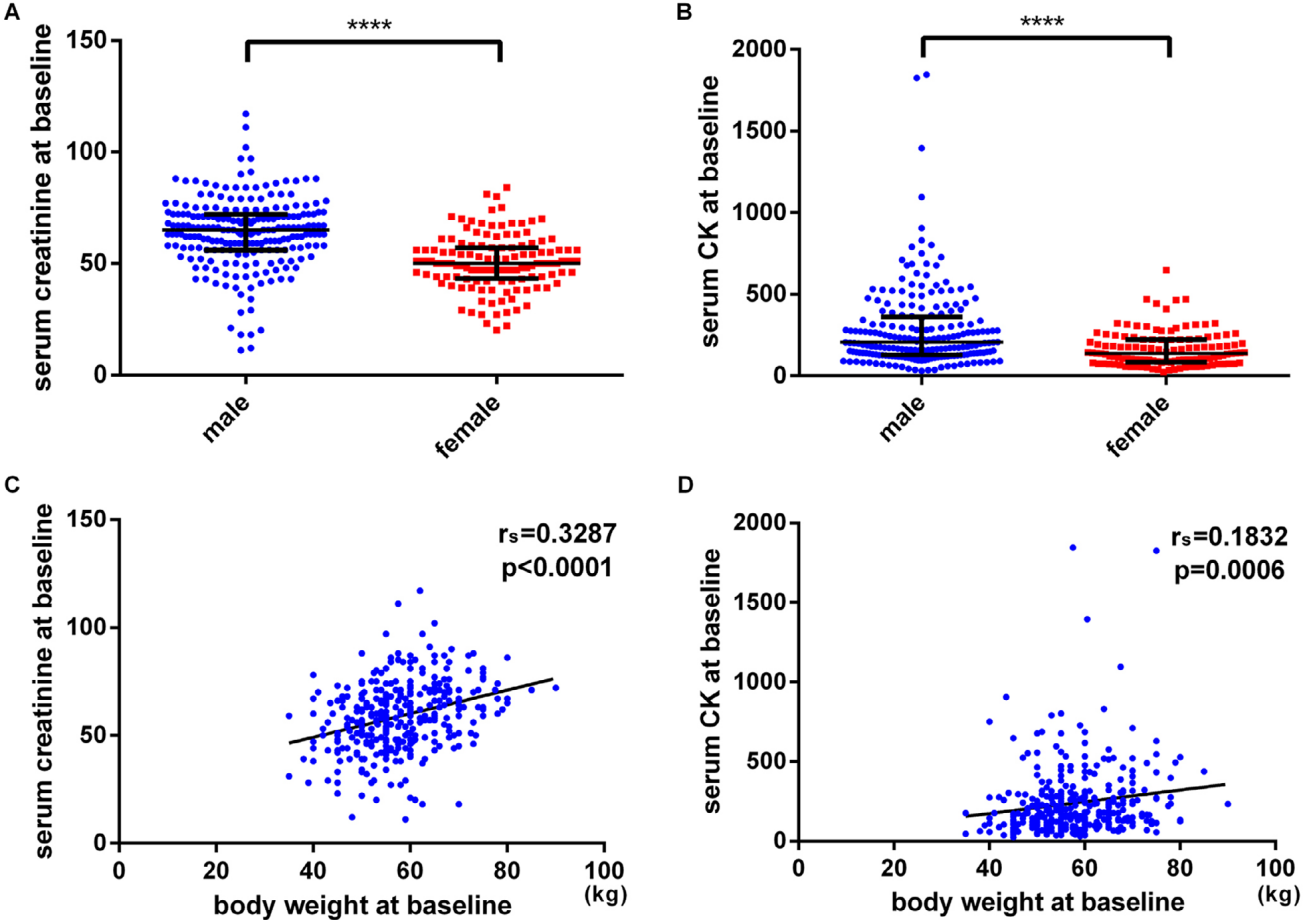
(A) Distribution analysis of baseline serum creatinine levels in male and female ALS patients. (B) Distribution analysis of baseline serum CK levels in male and female ALS patients. (C) Correlation analysis between serum creatinine levels and body weight. (D) Correlation analysis between serum CK levels and body weight. **** indicated *P* < 0.0001.

A total of 346 ALS cases with serum creatinine and CK measurements were enrolled serially, and they were followed up more than 12 months. There were 218 males and 128 females (ratio of male to female was 1.7:1). Sixteen patients (4.6%) had a positive family history of ALS, and the remaining 330 cases were sporadic. The mean age of disease onset was 55.5 ± 11.2 years old. According to the site of onset, 76 cases were bulbar‐onset, 173 cases were upper limb‐onset, and 97 cases were lower limb‐onset. The median diagnostic delay was 11.0 months (IQR: 6.0, 16.0). The median ALSFRS‐R score at baseline was 41 (IQR: 38, 44). The median serum creatinine level at baseline was 59 *μ*mol/L (IQR: 48.8–69). The median serum CK level at baseline was 174.5 U/L (IQR: 106.25–286.75). The detailed clinical features were showed in Table 1. We further analyzed the distribution of serum creatinine levels among male and female patients, and it showed that the male patients had higher creatine concentrations than that in female patients (63.8 ± 15.8 vs. 50.4 ± 12.4 *μ*mol/L; *P* < 0.0001; Fig. 1A). Similarly, higher serum CK levels were observed in male patients than that in female patients, and the median CK levels were 205.5 and 137.0 U/L, respectively (*P* < 0.0001; Fig. 1B). Besides, both serum creatinine and CK levels were correlated with body weight (Fig. 1C and D). The average concentrations of creatinine in King’s College stage 1, stage 2, and stage 3 + 4 were 63.7, 57.7, and 52.9 *μ*mol/L, respectively (*P* < 0.0001, Fig. S1A). The average concentrations of CK in stage 1, stage 2, and stage 3 + 4 were 245, 265, and 194 U/L, respectively (*P* = 0.0709, Fig. S1B).

**Table 1:** Clinical characteristic of patients with ALS.

Variables and subgroups	No.(%), mean ± SD or median (IQR)
Gender	
Male	218 (63.0%)
Female	128 (37.0%)
Family history	
Yes	16 (4.6%)
No	330 (95.4%)
Age of onset, mean ± SD,y	55.5 ± 11.2
Site of onset	
Bulbar	76 (22.0%)
Upper limb	173 (50.0%)
Lower limb	97 (28.0%)
Diagnostic categories	
Definite	203 (58.7%)
Probable	95 (27.4%)
Possible	48 (13.9%)
Diagnostic delay, median (IQR), mo	*n* = 346, 11.0 (6.0, 16.0)
BMI at baseline, mean ± SD, kg/m2	*n* = 346, 21.52 ± 2.81
ALSFRS‐R score at baseline, median (IQR)	*n* = 346, 41 (38, 44)
ΔALSFRS‐R, median (IQR)	*n* = 346, 0.67 (0.36, 1.20)
Use of riluzole	
Yes	245 (70.8%)
No	101 (29.2%)
PEG	
Yes	30 (8.7%)
No	316 (91.3%)
NIPPV	
Yes	52 (15.0%)
No	294 (85.0%)
Serum creatinine at baseline, median (IQR), *μ*mol/L	*n* = 346, 59.0 (48.8, 69.0)
Serum CK at baseline, median (IQR),U/L	*n* = 340, 174.5 (106.25, 286.75)

Abbreviations: ALS, amyotrophic lateral sclerosis; ALSFR‐R, Amyotrophic Lateral Sclerosis Functional Rating Scale–Revised; BMI, body mass index; CK, creatine kinase;IQR, Interquartile range; NIPPV, non‐invasive positive pressure ventilation; PEG, percutaneous endoscopic gastrostomy; SD, Standard deviation.

### Decreased serum creatinine levels linked with higher mortality risk

In this cohort of ALS patients, there were eight cases simultaneously combining with cancers, including two cases with breast cancer, two cases with lung cancers, one case with hepatic carcinoma, one case with nasopharyngeal carcinoma, one case with glioma, and one case with rectal cancer. Additionally, eight patients were lost to follow‐up. The remaining 330 cases were recruited to the final survival analysis. There were 236 (71.5%) patients reached the combined endpoint, among them 219 cases were deceased and 17 cases underwent tracheotomy. The median time from first symptom onset to death or tracheotomy of all the cases was 35 months.

**Figure 2:**
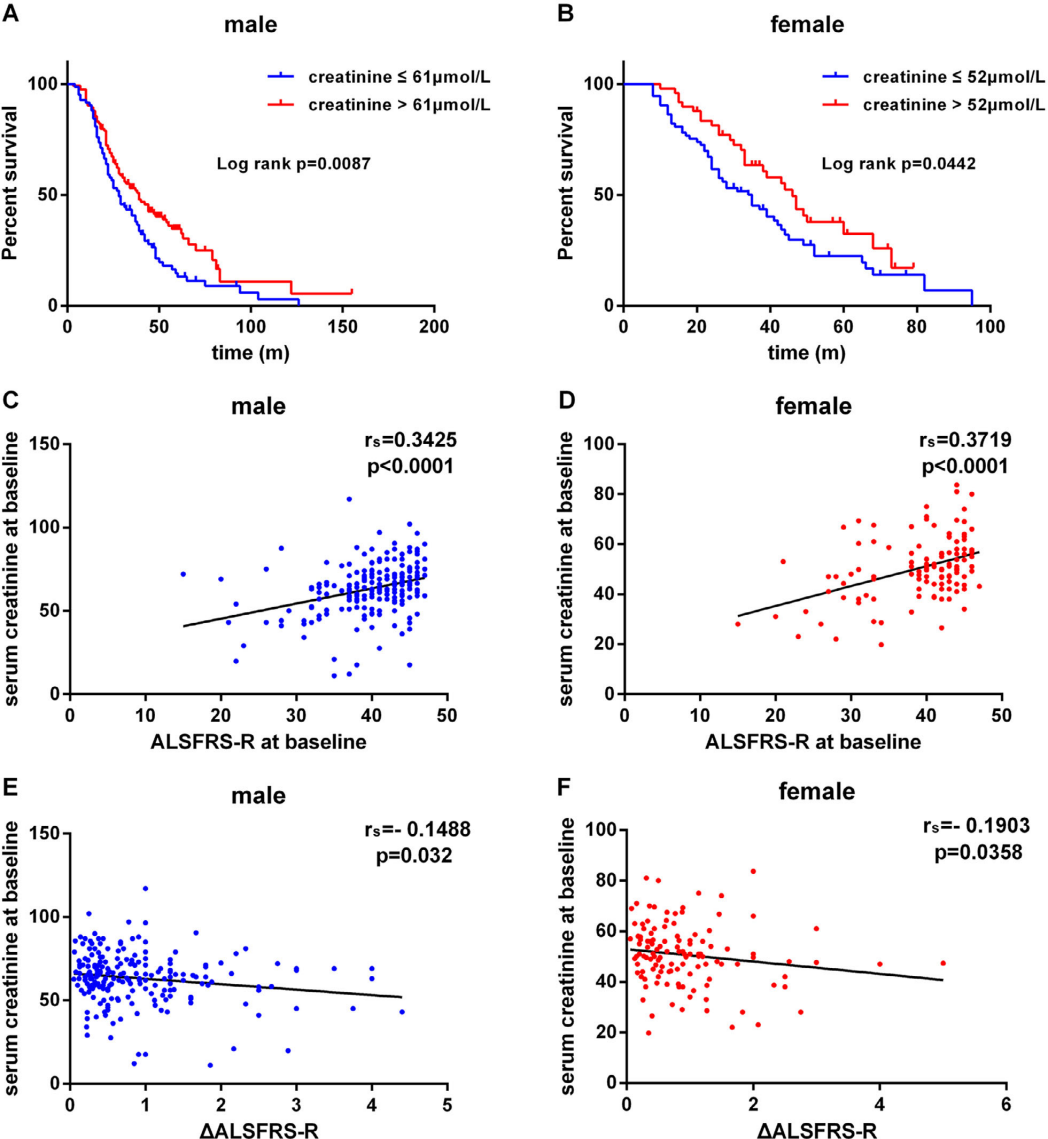
(A) Survival curves for male patients with creatinine ≤61 *μ*mol/L vs. creatinine > 61 *μ*mol/L. (B) Survival curves for female patients with creatinine ≤ 52 *μ*mol/L vs. creatinine > 52 *μ*mol/L. (C) Correlation analysis between serum creatinine levels and ALSFRS‐R scores in males. (D) Correlation analysis between serum creatinine levels and ALSFRS‐R scores in females. (E) Correlation analysis between serum creatinine levels and ΔALSFRS‐R in males. (F) Correlation analysis between serum creatinine levels and ΔALSFRS‐R in females.

Given the significantly different distribution of serum creatinine and CK levels among the male and female patients, we performed the subgroup survival analysis based on gender. The cut‐off values of male creatinine levels, female creatinine levels, male CK levels, and female CK levels were 61 *μ*mol/L, 52 *μ*mol/L, 177 U/L, and 174.5 U/L, respectively, which were identified using the X‐tile software. Among the male ALS patients, the median survival for higher creatinine group (>61 *μ*mol/L) and lower creatinine group (≤61 *μ*mol/L) was 39 months and 28 months, respectively (Log rank test, Chi square = 6.876, *P* = 0.0087; Fig. 2A). Among the female ALS patients, the median survival for higher creatinine group (>52 *μ*mol/L) and lower creatinine group (≤52 *μ*mol/L) was 46 months and 35 months, respectively (Log rank test, Chi square = 4.047, *P* = 0.0442; Fig. 2B). However, the serum CK levels were not associated with the survival in both male and female ALS patients (Fig. S2A and B).

Finally, multivariable Cox regression models were employed to identify the survival‐related variables. Among the males, a shorter diagnostic delay (≤12 m, HR: 2.269; 95%CI: 1.512–3.405), lower BMI (≤18.70 kg/m^2^, HR: 3.013; 95%CI: 1.951–4.652), faster disease progression (ΔALSFRS‐R > 0.63, HR: 3.699; 95%CI: 2.456–5.571), and lower creatinine levels (≤61 *μ*mol/L, HR: 1.629; 95%CI: 1.168–2.273) were associated with a short survival (Table 2). Among the females, an older age of onset (>55 years, HR: 2.515; 95%CI: 1.534–4.121), shorter diagnostic delay (≤12 m, HR: 2.653; 95%CI: 1.594–4.414), lower BMI (≤18.70 kg/m^2^, HR: 2.277; 95%CI: 1.200–4.321), faster disease progression (ΔALSFRS‐R > 0.63, HR: 2.984; 95%CI: 1.726–5.158), and lower creatinine levels (≤52 *μ*mol/L, HR: 1.677; 95%CI: 1.042–2.699) were associated with a high mortality risk (Table 2). We also assessed the effect of log CK on the survival outcome, and it showed that the baseline log CK was not associated with survival after multivariable Cox regression analysis (For male: HR: 1.062, 95%CI: 0.652–1.729, *P* = 0.808; For female: HR: 1.165, 95%CI: 0.489–2.774, *P* = 0.730.). Besides, in both male and female patients, the baseline serum creatinine levels were positively associated with disease severity (expressed as ALSFRS‐R score; Fig. 2C and D), and inversely associated with disease progression rate (expressed as ΔALSFRS‐R; Fig. 2E and F).

**Table 2:** Multivariable Model in Male and Female using multivariable Cox survival analysis (Backward Stepwise, Wald) after adjustment for possible influencing factors.^1^

Variables and subgroups	*β*	SE	Wald	*P*‐value	HR (95%CI)
Male					
Diagnostic delay					
>12 m					1.0 [Reference]
≤12 m	0.819	0.207	15.642	<0.001	2.269 (1.512, 3.405)
BMI at baseline					
>18.70					1.0 [Reference]
≤18.70	1.103	0.222	24.767	<0.001	3.013 (1.951, 4.652)
ΔALSFRS‐R					
≤0.63					1.0 [Reference]
>0.63	1.308	0.209	39.198	<0.001	3.699 (2.456, 5.571)
Creatinine at baseline					
>61 *μ*mol/L					1.0 [Reference]
≤61 *μ*mol/L	0.488	0.17	8.253	0.004	1.629 (1.168, 2.273)
Female					
Age of onset					
≤ 55y					1.0 [Reference]
>55 y	0.922	0.252	13.387	<0.001	2.515 (1.534, 4.121)
Diagnostic delay					
>12 m					1.0 [Reference]
≤12 m	0.976	0.26	14.101	<0.001	2.653 (1.594, 4.414)
BMI at baseline					
>18.70					1.0 [Reference]
≤18.70	0.823	0.327	6.347	0.012	2.277 (1.200, 4.321)
ΔALSFRS‐R					
≤0.63					1.0 [Reference]
>0.63	1.093	0.279	15.314	<0.001	2.984 (1.726, 5.158)
Creatinine at baseline					
>52 *μ*mol/L					1.0 [Reference]
≤52 *μ*mol/L	0.517	0.243	4.526	0.033	1.677 (1.042, 2.699)

^1^ Possible influencing factors included age of onset, site of onset, diagnostic delay, BMI, ALSFRS‐R score, progression rate (ΔALSFRS‐R), use of riluzole, use of NIPPV, and acceptance of PEG.

### Alteration of serum creatinine levels over time

**Figure 3:**
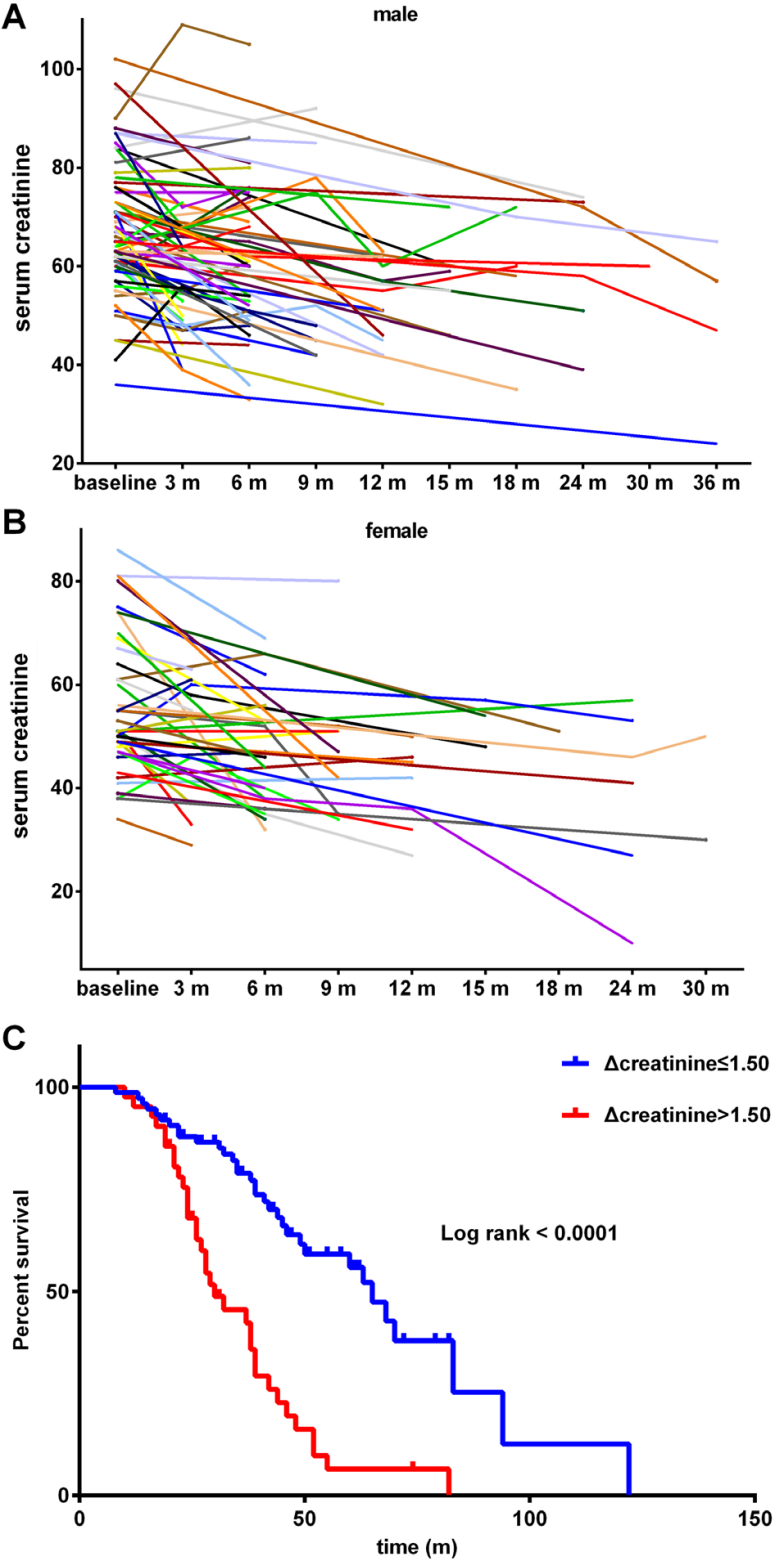
(A) The line chart of creatinine levels for male patients at different time points of follow‐up. (B) The line chart of creatinine levels for female patients at different time points of follow‐up. (C) Survival curves for creatinine slope of decline >1.5 vs. ≤1.5.

During follow‐up, 117 patients accepted repeated serum creatinine and CK measurements, and the distribution of creatinine and CK levels at baseline and at different time points of follow‐up was shown in Figure 3A and B and Figure S3A and B. The concentrations of CK were highly variable, while, serum creatinine levels tended to be decreased along with the disease progression. The association between declines of creatinine with survival was further analyzed. The decline in creatinine (Δcreatinine) was calculated by the formula: (creatinine at baseline – creatinine at first time of follow‐up) /months between follow‐up and baseline. The Δcreatinine for all patients ranged from −6.37 to 11.35. The higher creatinine slope of decline (>1.5) showed shorter median survival, compared to the lower group (≤1.5) (30.0 months vs. 65.0 months, Chi square = 28.25, *P* < 0.0001; Fig. 3C).

## Discussion

For ALS, there is an urgent need to identify a reliable and easily detectable prognostic biomarker, which will largely facilitate the assessment of disease progression and therapeutic effect in pharmacological trials. Serum creatinine and CK are two easily available chemical markers of muscle metabolism, which are strongly correlated with muscle mass and muscle loss.^5^, ^6^, ^15^ The aim of this study was to determine the value of serum creatinine and CK in monitoring disease progression and predicting survival in a prospective cohort of ALS patients. We observed that serum creatinine, not CK, was an independent predictive factor for survival in both male and female ALS patients, even after multiple adjustments of BMI, age of onset, site of onset, ALSFRS‐R score, progression rate (ΔALSFRS‐R), diagnostic delay, and use of riluzole. Moreover, the slope of creatinine decline was also predictive of survival of ALS.

Serum creatinine is a breakdown product of creatine phosphate in muscle. The neuroprotective effects of creatinine have been identified in transgenic mice model of ALS, with significant improvement in motor performance and extension of survival.^7^, ^8^ Chen et al. also observed that the sporadic ALS patients had significantly lower serum creatinine levels compared to the age‐ and gender‐matched healthy controls.^9^ Regarding the value of serum creatinine in prediction of ALS survival, we found that higher baseline creatinine levels were predictive of longer survival in both male and female ALS patients, which was similar to previous studies.^10^, ^11^ However, a deleterious impact of serum creatinine on survival has not been found in another cohort of sporadic ALS patients from West of China.^9^ In the present study, we also observed that the serum creatinine was positively correlated with the ALSFRS‐R score, and inversely linked to disease progression rate (Δ ALSFRS‐R), which was similar to the result from a meta‐analysis.^12^ Besides, Mitsumoto et al. also found that the plasma creatinine predicted ALS survival the best, compared to the oxidative stress biomarkers, including plasma uric acid, urinary 8‐oxo‐deoxy guanosine (8‐oxodG), and 15‐F2t‐isoprostane (IsoP).^16^ The application of creatinine as an outcome in clinical trial could significantly reduce the sample size by 21.5%.^10^ Taken together, we provided additional evidence that serum creatinine could be an inexpensive and easily accessible prognostic and survival‐related biomarker for ALS, which has the potential to be widely applied in clinical trials.

In ALS patient, a normal to mild elevation of serum CK level is frequently observed, which has been proved to be connected with lower motor neuron loss and muscle denervation.^15^, ^17^ However, the role of CK in the prediction of disease progression was inconsistent, and the ALS patients showed a wide distribution of serum CK levels. Gibson et al. performed a 48‐week non‐interventional longitudinal study for 80 ALS patients, and they observed that about 45% of patients had at least one high CK value during disease course, while there was no trend of high CK level over the study period.^6^ Prior et al. reported a 58‐year‐old male ALS patient, whose serum CK was 353 U/L at the time of diagnosis and sharply raised to 1905 U/L 6 months later.^18^ Rafiq and colleagues reported that higher CK enzyme levels were associated with longer survival in ALS.^19^ On the contrary, Gibson et al. observed that lower CK levels were correlated with better outcome with a longer survival.^6^ In this study, we found that the male ALS patients harbored significant higher levels of serum CK than female patients; however, the baseline CK levels were not significantly associated with survival, which was consistent with previous studies.^20^, ^21^

In the present study, we also noted some limitations. Firstly, 16 familial cases were included in this cohort study; however, we have not carried out the subgroup analysis based on family history, due to a small sample size from a single medical center. Secondly, a small portion of serum creatinine and CK measurements was performed in other medical centers, leading to the variable of normal range of serum creatinine and CK. Finally, there were other survival‐related factors which have not been included in our study, for instance, the pulmonary function parameters and serum urate.

To sum up, the male ALS patients have significantly higher levels of serum creatinine and CK than female patients. There is a positive correlation between baseline serum creatinine levels and ALSFRS‐R scores, and an inversely link with disease progression rate. Serum baseline creatinine levels showed strong correlation with mortality risk in both male and female ALS patients, and lower creatinine levels could predict poorer prognosis and shorter survival. In this cohort of ALS patients, a deleterious impact of serum CK on survival has not been identified. Serum creatinine is a reliable and easily accessible biomarker of disease severity in ALS and could be used in defining disease prognosis at the time of diagnosis.

## Conflicts of Interest

None.

## Author Contributions

Study concept and design (N Wang and Q‐J Zhang); acquisition and interpretation of data (Q‐F Guo, W Hu, L‐Q Xu and L Hao); drafting of the manuscript (Q‐J Zhang, Q‐F Guo and W Hu); critical revision of the manuscript for important intellectual content (N Wang and Q‐J Zhang); obtaining of funding (Q‐J Zhang and N Wang); study supervision (Q‐J Zhang and N Wang).

## Supporting information


**Figure S1.** The concentrations of serum creatinine (A) and CK (B) in ALS patients with different King’s College stages.
**Figure S2.** (A) Survival curves for male patients with CK ≤ 177 U/L vs. CK > 177 U/L. (B) Survival curves for female patients with CK ≤ 174.5 U/L vs. CK > 174.5 U/L.
**Figure S3.** (A) The line chart of CK levels for male patients at different time points of follow‐up. (B) The line chart of CK levels for female patients at different time points of follow‐up.
