# Supplementary material for: Decreased serum creatinine levels predict short survival in amyotrophic lateral sclerosis
Source: Ann Clin Transl Neurol. 2021 Jan 15;8(2):448–55. doi: 10.1002/acn3.51299 (PMC7886033; doi:10.1002/acn3.51299)
Supplement: Supplementary file 1 — Figure S1. The concentrations of serum creatinine (A) and CK (B) in ALS patients with different King’s College stages. Figure S2. (A) Survival curves for male patients with CK ≤ 177 U/L vs. CK > 177 U/L. (B) Survival curves for female patients with CK ≤ 174.5 U/L vs. CK > 174.5 U/L. Figure S3. (A) The line chart of CK levels for male patients at different time points of follow‐up. (B) The line chart of CK levels for female patients at different time points of follow‐up. [file ACN3-8-448-s001.docx]

**Title:** Decreased serum creatinine levels predict short survival in amyotrophic lateral sclerosis

**Authors:** Qi-Fu Guo, Wei Hu, Liu-Qing Xu, Hao Luo, Ning Wang, Qi-Jie Zhang


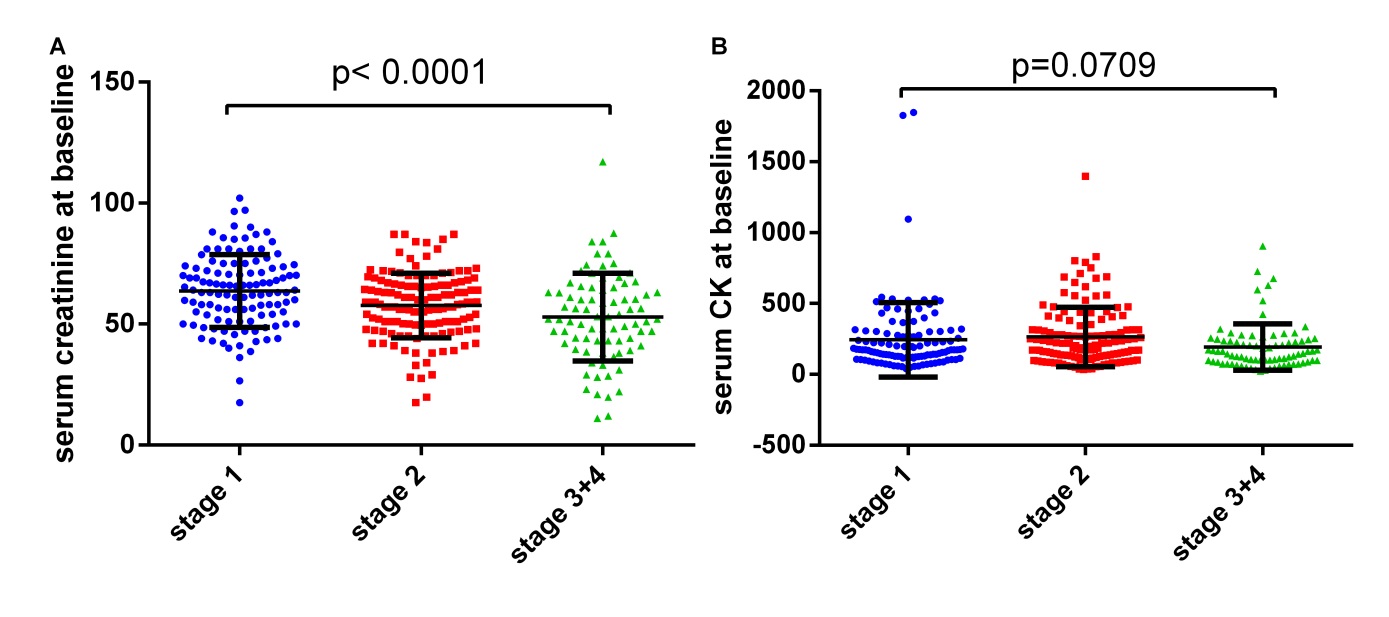


Supplemental Figure 1. The concentrations of serum creatinine (A) and CK (B) in ALS patients with different King’s College stages.


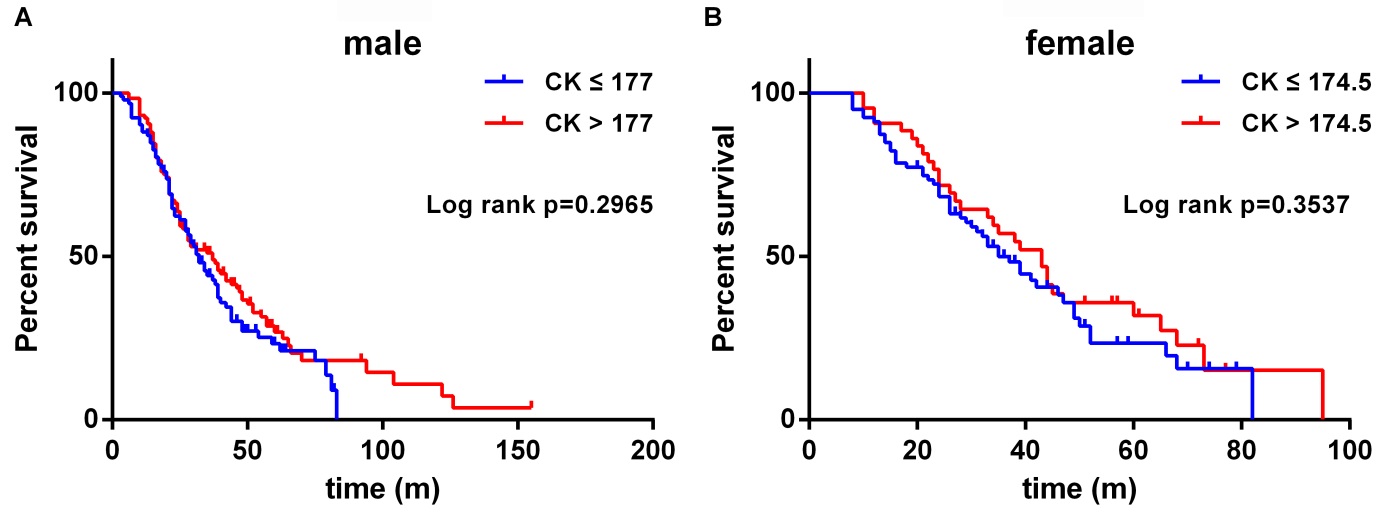


Supplemental Figure 2. (A) Survival curves for male patients with CK ≤177 U/L vs. CK >177 U/L. (B) Survival curves for female patients with CK ≤174.5 U/L vs. CK >174.5 U/L.


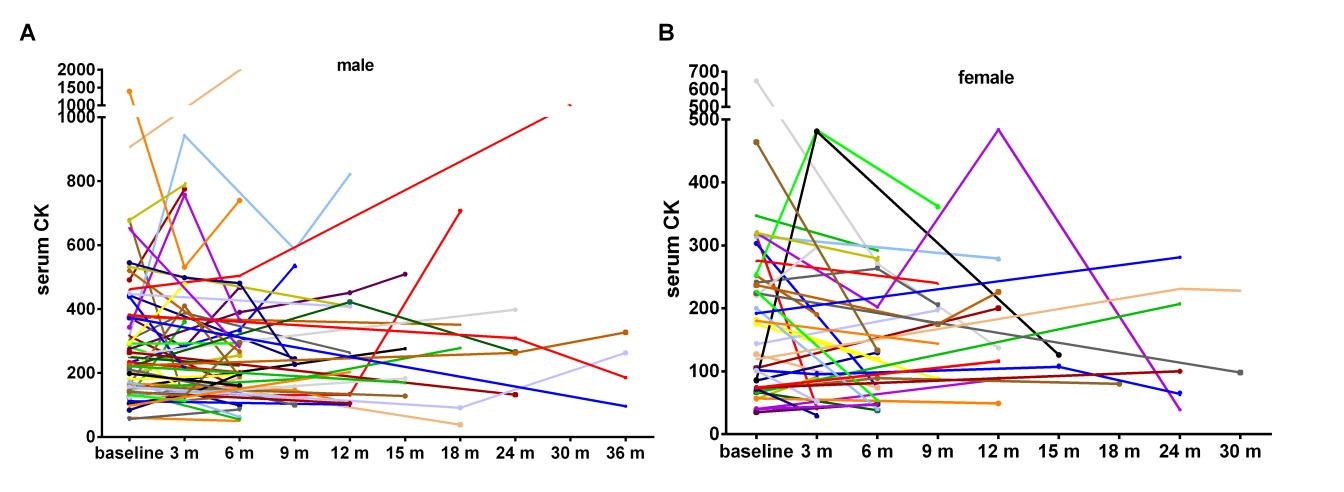


Supplemental Figure 3. (A) The line chart of CK levels for male patients at different time points of follow-up. (B) The line chart of CK levels for female patients at different time points of follow-up.
